# Communication of Diagnosis of Infertility: A Systematic Review

**DOI:** 10.3389/fpsyg.2021.615699

**Published:** 2021-03-18

**Authors:** Laura Mosconi, Giada Crescioli, Alfredo Vannacci, Claudia Ravaldi

**Affiliations:** ^1^CiaoLapo Foundation for Perinatal Health, Prato, Italy; ^2^Perinatal Research Laboratory (PeaRL), University of Florence, Florence, Italy; ^3^Section of Pharmacology and Toxicology, Department of Neurosciences, Psychology, Drug Research and Child Health, University of Florence, Florence, Italy

**Keywords:** infertility, care, communication, counseling, prenatal care, perinatal care

## Abstract

**Background:** When infertility is diagnosed, physicians have the difficult task to break bad news. Their communication skills play a central role in improving patients' coping abilities and adherence to infertility treatments. However, specific guidelines and training courses on this topic are still lacking. The aim of the present study is to provide some practical advice for improving breaking bad news in infertility diagnosis through a systematic literature review of qualitative and quantitative studies.

**Methods:** Electronic searches were performed in the MEDLINE, Embase, PsycINFO, Cumulative Index to Nursing and Allied Health Literature (CINAHL), and Psychology and Behavioral Sciences Collection databases. All articles focusing on the communication of the diagnosis of infertility were included. The main findings of each included article were then summarized.

**Results:** Literature search identified 11,838 references that were screened for eligibility. Full texts of 81 articles were retrieved, and their analysis led to the inclusion of 4 articles, which treated the theme of communication of infertility only partially. The main addressed aspects concerning the communication of the infertility diagnosis were the following: (i) the value that patients give to healthcare professionals' communication skills; (ii) the importance of giving clear information on diagnostic procedures and treatments in order to decrease patients' anxiety; and (iii) the importance of involving both partners.

**Conclusions:** This review pointed out that the communication of the infertility diagnosis is still underinvestigated. Specific guidelines are currently not available, but other protocols could be used. Taking into account the principal aspects of communication highlighted with this review, in this study, we suggested an adaptation of the original SPIKES protocol that could be used by healthcare professionals for the communication of the infertility status.

## Introduction

Infertility is an extensive problem worldwide. It has been estimated that in 2010, there were 48.5 million infertile couples all over the world. Around 2 out 100 women between 24 and 44 years old suffer from primary infertility, while 10 out 100 women suffer from secondary infertility (Mascarenhas et al., 2012). In Italy, the “Istituto Superiore di Sanità” estimates that around 15% of couples suffer from infertility (Istituto Superiore di Sanità). Most couples begin to fear an infertility issue after a few months of unsuccessful attempts to conceive. The time frame between the decision to have a child and the diagnosis of infertility is very stressful: infertility is a physical condition that has a direct impact on the individual's perception of physical integrity, on the couple functioning, and on the couple's short- and long-term life projects (Ansha Patel and Sharma, 2018). For this reason, the diagnosis of infertility has a strong impact on women' and couples' well-being. In a study of Domar et al., about 500 women with several medical conditions completed the Symptom Checklist-90-Revised (SCL-90R). The results suggest that the psychological impact of stress related to reproductive problems could be comparable to those of other long-term medical conditions such as cancer, undergoing cardiac rehabilitation, and hypertension (Domar et al., 1993).

When infertility is diagnosed, physicians have the difficult task to break bad news. However, little is known about communication in this field. The diagnosis of infertility has a strong impact *per se*, and it could be defined a “symbolic loss” and is related to an “infertility grief.” The “symbolic loss” related to the diagnosis of infertility is not clear and visible to others, while other life events are clear and identifiable forms of loss, such as the death of a loved one. In other conditions, the loss is publicly recognized, and the bereaved are likely to receive support throughout their mourning. They can openly discuss their feelings of loss, and the grieving process follows cultural norms that include rituals to mitigate the grieving process (McBain and Reeves, 2019). None of this happens following a diagnosis of infertility.

Moreover, infertility diagnosis is related to many other challenges for couples: they have to decide the subsequent steps, and they have to discuss the risks and limitations related to infertility treatments with healthcare professionals. This has a deep impact on their health and quality of life: according to a literature review, women who received a diagnosis of infertility had significantly lower scores on mental health, social functioning, and emotional behavior (Chachamovich et al., 2010). It should be taken into account that infertility treatments have a poor outcome for most couples. In fact, in Italy, the percentage of live births with intrauterine insemination (IUI) over the total of assisted reproductive technology (ART) cycles is 6.9%, while with FIVET or intracytoplasmatic sperm injection (ICSI) is 11.3% (Istituto Superiore di Sanità-ISS, 2017). In this sense, sometimes, the diagnosis of infertility is not the only and last piece of bad news: for instance, people who resort to ART often receive further bad news during the diagnostic workup and the infertility treatment (reiteration of bad news) (Lalos, 1999; Leone et al., 2017). It is useful to remember that bad news following the primary diagnosis of infertility is one of the reasons for patients' dropout before completing infertility treatments; moreover, poor management of psychological aspects is listed among the main causes of treatment discontinuation (Gameiro et al., 2012). Quality of communication is a key point for improving patients' coping abilities, well-being, adherence to infertility treatments, and patient–provider continuity of care. The latter is one of the main factors of patient-centered care (PCC) that could prove essential for treatment compliance (Palmer-Wackerly et al., 2019). However, while patients claim for clear information, honesty, emotional support, and respect (Ussher et al., 2018), healthcare providers should have access to adequate training programs. Some experiences demonstrated that nurses' knowledge of reproductive issues, communication skills, and practice behaviors increased significantly after structured courses (Quinn et al., 2019).

Nowadays, the impact of breaking bad news on healthcare providers and their perceptions in the relationship with the patient are still poorly characterized. The fear of inflicting pain or not to fully understand patients' discomfort, lack of time, and the complex management of patients' expectations are just some of the problems identified (Klitzman, 2018). In addition, more attention must be paid to psychological care after the diagnosis of infertility and after the subsequent bad news due to the failure of ART (e.g., a negative pregnancy test). In particular, the current literature highlights the importance of specific psychological interventions to reduce stress and to improve couples' well-being.

Although there is a proposal of guidelines on how to communicate bad news during ART (based on SPIKES protocol) (Leone et al., 2017), shared protocols and guidelines on how to communicate the diagnosis of infertility are currently not available. The fields of infertility and ART are strongly connected but show different communication issues. As mentioned above, in the context of ART, there is a reiteration of bad news connected to repeated treatment failures and the clinical ineffectiveness of medical treatments (Leone et al., 2017). Meanwhile, infertility diagnosis involves couples before the beginning of the ART path. In this case, they face bad news for the first time and have not dealt with an alternation of hope and despair. For this reason, the aim of the present systematic literature review of qualitative and quantitative studies is to explore existing research focusing on the communication of the diagnosis of infertility and to highlight existing evidence on physician–patient relationship in this field. Starting from this point, the final goal of our research was to provide some practical advice for improving breaking bad news in clinical practice.

## Materials and Methods

### Search Strategy

Data for the systematic review were obtained through a search strategy based on the intersection of two main domains. The first one was related to infertility, communication, and physician–patient relationship. The second one focused on healthcare professionals vs. patients (women and couples). Electronic searches were performed in the MEDLINE, Embase, PsycINFO, Cumulative Index to Nursing and Allied Health Literature (CINAHL), and Psychology and Behavioral Sciences Collection (PBSC) databases. Complete search strategies for all databases are provided within the Supplementary Material 1.

### Inclusion and Exclusion Criteria

We included all studies, posters, and abstracts published in English or Italian in scientific journals between January 2000 and March 5, 2020. We included all qualitative and quantitative studies, independently from their study design, containing information about breaking bad news in infertility. Additional searches in the reference lists of retrieved manuscripts were also performed. We excluded all papers that deal with infertility care but did not mention how to communicate the diagnosis of infertility. We also excluded remaining articles concerning patients' coping strategies and psychological adaptation after receiving the diagnosis of infertility.

### Selection Process and Data Extraction

Records were retrieved on the same day from all sources. Two investigators (CR, LM) independently selected the studies (double-blind selection). Discrepancies about inclusion/exclusion were resolved through discussion or in consultation with a third reviewer (AV, GC). CR and LM reviewed the main reports and Supplementary Material and extracted all relevant information for the included studies. In case of doubt or missing information, we contacted the authors of the original paper. For each paper, the following qualitative and/or quantitative data were extracted: country, type of study (i.e., quantitative, qualitative, guidelines), perspective [i.e., patients' or healthcare practitioners' (HCPs')], main results, and parameters used by authors for their evaluation (i.e., theme for qualitative studies, scores for quantitative studies), and main results concerning communication and relationship with the staff.

### Quality of Included Studies

Quality of included studies was evaluated using the Checklist for Qualitative Research by the Joanna Briggs Institute (JBI) (JBI Critical Appraisal Checklist for Qualitative Research, 2020) and with the Checklist for the Quality Assessment of Guidelines (AGREE II) (Canadian Agency for Drugs and Technologies in Health (CADTH), 2014).

This systematic review was reported in accordance with the preferred reporting items for systematic reviews and meta-analyses (PRISMA) (Moher et al., 2009).

## Results

**Figure 1:**
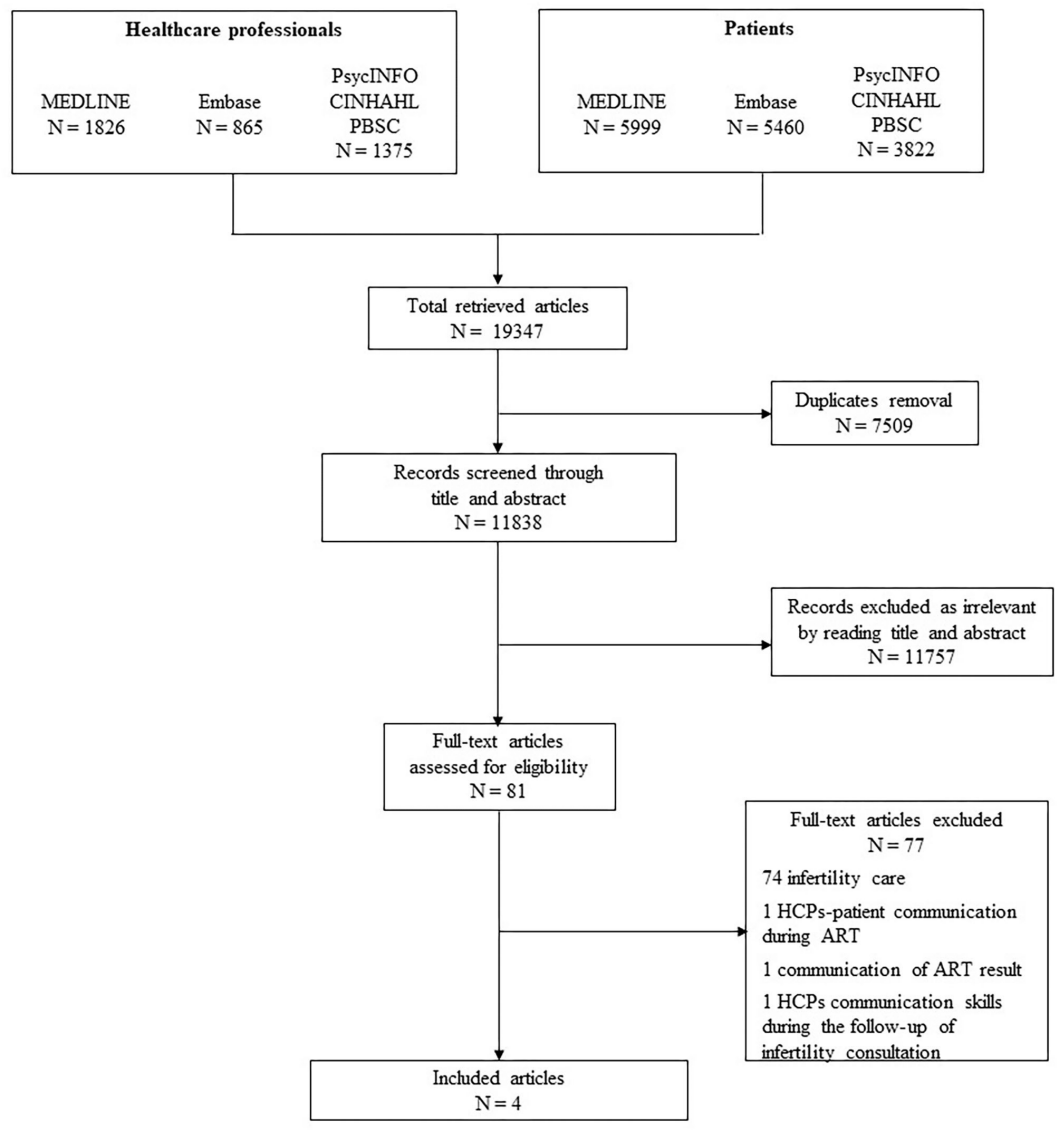
Preferred reporting items for systematic reviews and meta-analyses PRISMA flowchart of search strategy results.

A flow chart describing the results of the selection process is reported in Figure 1. The literature search identified 19,347 references. After removing duplicates (*n* = 7,509), titles and abstracts of 11,838 references were screened. Of those, 11,757 were excluded. We retrieved the full texts of the remaining 81 references and assessed them for eligibility.

Seventy-seven studies were excluded due to the absence of information about breaking bad news in infertility. These papers deal with infertility care, but there was no reference to the specific topic explored in the present review (communication of the infertility diagnosis). For instance, the paper “Impediments to communication and relationships between infertility care providers and patients” concerns communication issues between ART practitioners and patients that underwent infertility treatment (Klitzman, 2018). However, it does not take into account how to communicate the diagnosis of infertility. Another study concerns the communication of results after the first cycle of ART, but it does not focus on giving bad news specifically and does not deal with the diagnosis of infertility itself (Groh and Wagner, 2005). A paper published in 2005 (Leite et al., 2005) focused on women's satisfaction with physicians' communication skills during a follow-up infertility consultation at the initial phase of the infertility treatment. Although physicians' communication skills are mentioned in the study, there is no advice on how to communicate the diagnosis of infertility. Once again the main focus is on ART and infertility treatment in general.

Ultimately, four studies were eligible according to the inclusion criteria (Dancet et al., 2012; Gameiro et al., 2015; Jafarzadeh-Kenarsari et al., 2015; Liu, 2015). Features of the analyzed studies are reported in Table 1. Two of them are qualitative studies, and the others are a poster presentation and a clinical guideline for psychosocial care in infertility and the medically assisted reproduction setting. Apart from one paper that has a mixed perspective of patients and HCPs (Jafarzadeh-Kenarsari et al., 2015), the others take only the patients' perspective. The main results of each paper are highlighted, with particular attention to the aspects of communication and relationship with staff. In particular, Dancet et al. (2012) suggested that patients “valued staff with skill in communicating bad news.” They reported no other advice concerning how to communicate the diagnosis of infertility. The study mainly concerns the “patient centered infertility care” model (PCIC) from the patients' perspective and does not explain its dimensions thoroughly. Authors gave just a brief description of them (Dancet et al., 2012). The European Society of Human Reproduction and Embryology (ESHRE) guidelines (Gameiro et al., 2015) pointed out that fertility staff should provide information about diagnostic procedures to decrease patients' anxiety and stress related to the process itself. Moreover, the authors recommended involving both partners during the diagnosis. These guidelines are a landmark in the infertility care field, but their primary focus is the treatment of infertility. Finally, the poster presentation by Liu (2015) suggested that Chinese patients valued receiving information about their diagnosis (Liu, 2015). Other advice concerned both the diagnosis and the treatment of infertility without a clear separation between the two steps. In particular, authors pointed out that women were more anxious to receive information than their male partners, while the latter dominated in clinical decision-making. Another paper from Iran highlights the patients' need to obtain comprehensive information about diagnosis and fertility treatment (Jafarzadeh-Kenarsari et al., 2015), and this is in line with the poster presentation by Liu (Liu, 2015). Inadequate knowledge about their condition leads to mistrust against HCPs as shown by a couple's words: “We are so unhappy because they refuse to explain what the problem is [...] we have to search the web to find some answers.” ^18^ Finally, authors of the ESHRE guidelines made a list of general principles of psychosocial care that patients value that could be applied to the communication of bad news (Table 2) (Gameiro et al., 2015). According to JBI's Checklist for the evaluation of the quality of included studies and according to the Checklist for the Quality Assessment of Guidelines (AGREE II), all papers show a good quality. However, the paper by Dancet et al. (2012) and the poster presentation by Liu (2015) do not mention any cultural or theoretical statement from the researchers, and they do not address any influence of the researcher on the research or vice versa. On the other hand, in the paper by Jafarzadeh-Kenarsari et al. (2015), the cultural and theoretical statement from the researchers is unclear, and the evaluation of the influence of the researcher on the research or vice versa are not applicable because it is part of a larger study. Although the poster presentation is not a qualitative study, data reported were evaluated even though we could not assess if the conclusion drawn in the study flowed from the analysis of the data. Moreover, the ESHRE guidelines show a high score in all domains except for the applicability domain where the average score is 4 in a scale from 1 to 7. The evaluation of the domain of “Editorial Independence” is not applicable because the guidelines were funded by the ESHRE group itself. Complete quality evaluation of included studies is available in Supplementary Material 2.

**Table 1:** Characteristic of included studies.

**References**	**Country**	**Type of study**	**Perspective**	**Patients**	**Main results of the study**	**Communication and relationship with staff**	**Quality of the study**
Dancet et al. (2012)	UK Spain Belgium Austria	Qualitative study	Patients' perspective	48 heterosexual patients (50% women) diagnosed with infertility and/or treated with IUI or IVF/ICSI	It has identified important specific care aspects about the 10 dimensions of “patient-centered infertility care:” provision of information, attitude of and relationship with staff, competence of clinic and staff, communication, patient involvement and privacy, emotional support, coordination and integration, continuity and transition, physical comfort, and accessibility.	Patients valued the following staff attitudes: being friendly, empathic, accessible, helpful, careful, respectful, and engaged. Patients appreciated being informed spontaneously in an understandable way (language, level) and valued staff with skill in communicating bad news.	This paper checks 8 out of 10 items of the scale.
Gameiro et al. (2015)	Europe	Guideline	Patients' perspective	–	Patients' preferences about psychosocial care and psychosocial needs, which can be behavioral (lifestyle, exercise, nutrition, compliance); relational and social (relationship with the partner, family, friends, work, and larger social networks), emotional (emotional well-being), cognitive (knowledge and concerns).	Patients valued: how staff relate to them, staff showing understanding and paying attention to the emotional impact of infertility, being involved in decision-making, sensitive and trustworthy staff members, minimal waiting times, not being hurried in medical consultation, continuity of care, receiving attention to their distinct needs related to their medical history, written information on treatment, explanations about treatment results and treatment options, understandable and customized (i.e., personally relevant) treatment information, and the provision of information about psychosocial care options.	It is evaluated with an overall score of 6 on a scale from 1 to 7.
Jafarzadeh-Kenarsari et al. (2015)	Iran	Qualitative study	Patients' and HCPs' perspective	26 infertile couples (17 men and 26 women) and 7 members of medical personnel (3 gynecologists and 4 midwives)	The study highlights part of couples' challenges and concerns, and necessity for cooperative assistance and support. Moreover, four main categories of infertile couples' needs are identified: infertility and social support, infertility and financial support, infertility and spiritual support, and infertility and informational support.	Patients underlined the importance of being informed on the disease (comprehensive information during diagnosis and treatment). Main problems encountered during the communication with HCPs: inadequate knowledge on the nature of the condition, the outcomes of a diagnostic and treatment method, and ignorant behavior of HCPs to patients' questions.	This paper checks 8 out of 10 items of the scale.
Liu (2015)	China	Poster presentation	Patients' perspective	200 infertile couples	The desire to receive information was significantly greater in female partners; male partners were more satisfied with information provision than female partners, the desire to participate in decision-making was greater in male than female partners, the desire to receive information and participate in decision-making was positively related to education.	Infertile couples were highly interested in receiving information about their diagnosis and treatment options and participating in clinical decision-making.	This poster presentation checks 7 out of 10 items of the scale.

*HCPs, healthcare professionals; ICSI, intracytoplasmatic sperm injection; IUI, intrauterine insemination; IVF, in vitro fertilization*.

**Table 2:** What fertility staff should be aware of about patients' needs.

**Infertility patients' needs**
How staff related to patients.
Staff should show understanding and pay attention to the emotional impact of infertility.
Patients need psychosocial care from sensitive and trustworthy staff members.
Patients want to receive attention to their specific needs related to their medical history.
Patients want minimal waiting time, continuity of care, and not hurried medical consultations.
Patients want personalized care and value professional competence of staff.
Patients want the opportunity to contact other patients.
Patients that express a need for emotional support value the opportunity to access specialized psychological interventions.
Positive staff characteristics (i.e., communication and respect) are associated with better patient well-being.

## Discussion

Infertility is a very common issue around the world, and it represents a milestone in a couple's life. Coping with the infertility status can be very complicated due to the sudden interruption of the family plan and the lack of acknowledgment of the couple's grief. However, HCPs can avoid further trauma and pain using good communication. How the diagnosis is communicated could improve patients' well-being and the ability to cope with it.

In this literature review, we did not find any protocol or guideline concerning breaking bad news in infertility. Many papers concern infertility treatment care or ART, for instance, the paper of Leone et al. (2017) about a proposal of guidelines about breaking bad news in ART. However, we found some useful information that could help practitioners in their daily practice whenever they face an infertility diagnosis. About this, Dancet et al. (2012) suggested that patients value staff's skills on communicating bad news^16^. This is in agreement to the ESHRE guidelines (Gameiro et al., 2015), which points out that patients value positive staff characteristics including communication skills, which are linked to the couples' well-being. Moreover, we should consider that some patients report unprofessional communication from HCPs. In fact, they point out that they felt the practitioners' fear during the diagnosis of infertility (Dancet et al., 2011).

Giving information is linked to the staff's communication skills, and it is very important to patients according to the ESHRE guideline (Gameiro et al., 2015), Liu's (2015) and Jafarzadeh-Kenarsari et al. (2015) works. Another important issue is the need for personalized, sensitive, and continuous care from trustworthy staff members who should show an understanding of the emotional impact of infertility (Gameiro et al., 2015). Communication, information, and continuity of care are three dimensions of PCC whose application is linked to the patients' well-being (Gameiro et al., 2013). PCC is usually valued more important by patients than HCPs (Van Empel et al., 2011), and this could be an obstacle to satisfy patients' needs. Moreover, giving poor information could lead to mistrust against HCPs (Jafarzadeh-Kenarsari et al., 2015), and it might result in inadequate care. Also, in this case, there is a different evaluation about the importance of information between patients and providers. The latter ones value providing information less important than patients do (Streisfield et al., 2015). This gap should be removed to improve the quality of care and to move from physician centered care to PCC.

Concerning psychological help and support, patients expect to have the possibility to access professional psychological care, and they want the opportunity to contact other couples (Gameiro et al., 2015). Patients have various counseling needs that HCPs should take into account and that involve several areas: emotional, sexual, marital, and the family one (Jafarzadeh-Kenarsari et al., 2015). Infertility counseling organizations agree that all patients who suffer from infertility should be able to access individual or couple counseling before, during, and after infertility treatment. Infertility counseling has different goals depending on the type of counseling itself: individual, couple, or group approach. Individual counseling allows exploring in greater depth concerns related to the experience and treatment of infertility, as well as coping mechanisms and social implications. Couple counseling enables patients to understand couple dynamics and to learn how to support each other. Finally, group counseling offers couples the opportunity to share their experience with others who are living in a similar situation (Van den Broeck et al., 2010). The literature helps mental health practitioners by showing the key issues that should be considered during infertility counseling (Stammer et al., 2002; Van den Broeck et al., 2010; Peterson et al., 2012). For instance, gender differences involve a diverse coping approach to the issue (Peterson et al., 2012).

Many other psychological approaches appear to be effective to reduce couples' stress and improve well-being. For instance, acceptance and commitment therapy helps patients to reduce stress and to increase couples' intimacy (Taheri et al., 2013). A paper about mindfulness-based cognitive approach points out how this technique can help to improve women's self-acceptance and their relationship with others (Fard et al., 2018).

### Implications for Clinical Practice

Although there is no specific guideline to communicate the diagnosis of infertility, other protocols currently adopted in daily medical practice could be used. SPIKES is an easy-to-follow protocol that has been used in the oncology setting for 20 years. It is divided in six steps, which help and facilitate HCPs to break bad news (Baile et al., 2000). These steps are identified as “Setting up, Perception, Invitation, Knowledge, Empathize, Strategy, and Summary.” SPIKES has been applied in the area of perinatal grief (Greiner and Conklin, 2015; Mosconi et al., 2019) and ART (Leone et al., 2017) and could be used to communicate the infertility status. In Table 3, we suggest an adaptation of the original SPIKES protocol for the infertility field, taking into account the results of this literature review. In particular, we modified some advice included in the original version of the SPIKES protocol, and we added other recommendations; for instance, we replaced the sentence “Manage time constraints and interruptions” with “Avoid interruptions and to be in a hurry during the consultation.” In fact, patients with infertility appreciate a thorough consultation without rush. These few actions could be very useful in clinical practice due to their easiness to be remembered and to be applied. Moreover, they could be integrated with the professional personal experience of HCPs.

**Table 3:** SPIKES protocol for infertility diagnosis.

**Phases**	**Actions**
Setting up	Try to minimize waiting time before the consultation.
	Arrange for some privacy.
	Involve both partners during the diagnosis.
	Sit down and try to not have barriers between you and patients.
	Make connection with the patients maintaining eye contact and/or touching them on the arm or holding a hand.
	Avoid interruptions and to be in a hurry during the consultation.
Perception	Try to understand what the patients know about their medical situation.
	Remember that patients value receiving attention to their specific needs related to their medical history.
Invitation	Try to understand how much information the patients want. Usually, patients with infertility appreciate knowing all information.
Knowledge	Use phrases to anticipate the bad news, for instance “I'm so sorry to tell you…” or “Unfortunately…”
	Avoid medical jargon
	Give comprehensive information.
Empathize	Try to pay attention to the emotional impact of the diagnosis.
	Remember that patients value a trustworthy and sensitive staff.
Strategy and summary	Ensure a continuity of care by planning follow-up with the same staff. Remember that patients value personalized care.
	Give the opportunity to contact other patients in a similar situation.
	Give the opportunity to access to a specialized psychological help.

## Strengths and Limitations

This review has several points of strength. First, the literature research was performed, retrieving articles from five large databases, and the literature screening was performed double blind, ensuring a rigorous methodology. Second, the extensive analysis of articles allowed us to highlight the lack of investigations focused on this topic and, therefore, to propose a new approach for the communication of infertility diagnosis. Finally, focusing on the importance of HCPs' communication skills in this field, this review may act as a starting point for future investigations and targeted interventions for HCPs.

The main limitation of this review lies in the scarcity of information reported in each of the included studies. In this light, the adaptation of the original SPIKES protocol may not include all aspects of patients' needs other than the few ones reported within the included articles. Further research could identify other areas of interest to be analyzed and included in an updated protocol for the communication of the infertility diagnosis.

## Conclusions

Most of the currently available literature is related to infertility treatment care, and its main focus was not the communication of infertility diagnosis, which represents the starting point of infertility grief for many couples. Only a few papers give some advice about breaking bad news, and there are no thorough guidelines about it. Based on the findings of this review, our adapted version of the SPIKES protocol is an easy-to-use guideline, which could be very useful for healthcare professionals and could be easily integrated in routinary clinical practices.

## Data Availability Statement

The original contributions presented in the study are included in the article/Supplementary Material, further inquiries can be directed to the corresponding author/s.

## Author Contributions

AV and CR: conceptualization and validation. GC and LM: original draft, methodology, writing, and formal analysis. All authors: writing, review, and editing.

## Conflict of Interest

The authors declare that the research was conducted in the absence of any commercial or financial relationships that could be construed as a potential conflict of interest.

## References

[B1] Ansha Patel P. S. V. N. Sharma P. K. (2018). In cycles of dreams, despair, and desperation: research perspectives on infertility specific distress in patients undergoing fertility treatments. J. Hum. Reprod. Sci. 11, 320–328. 10.4103/jhrs.JHRS_42_18 30787515 PMC6333040

[B2] Baile W. F. Buckman R. Lenzi R. Glober G. Beale E. A. Kudelka A. P. (2000). SPIKES—a six-step protocol for delivering bad news: application to the patient with cancer. Oncologist 5, 302–311. 10.1634/theoncologist.5-4-302 10964998

[B3] Canadian Agency for Drugs and Technologies in Health (CADTH) (2014). Stepwise Approach for the Prescription of Opiates for Non-Cancer Pain: A Review of Clinical Evidence and Guidelines. Available online at: https://www.ncbi.nlm.nih.gov/books/NBK263404/ (accessed December 01, 2020). 25520999

[B4] Chachamovich J. R. Chachamovich E. Ezer H. Fleck M. P. Knauth D. Passos E. P. (2010). Investigating quality of life and health-related quality of life in infertility: a systematic review. J. Psychosom. Obstet. Gynecol. 31, 101–110. 10.3109/0167482X.2010.481337 20443659

[B5] Dancet E. A. F. Dhooghe T. M. Sermeus W. Van Empel I. Strohmer H. Wyns C.. (2012). Patients from across Europe have similar views on patient-centred care: an international multilingual qualitative study in infertility care. Hum. Reprod. 27, 1702–1711. 10.1093/humrep/des061 22427309

[B6] Dancet E. A. F. Van Empel I. W. H. Rober P. Nelen W. L. D. M. Kremer J. A. M. Dhooghe T. M. (2011). Patient-centred infertility care: a qualitative study to listen to the patients voice. Hum. Reprod. 26, 827–833. 10.1093/humrep/der022 21317152

[B7] Domar A. D. Zuttermeister P. C. Friedman R. (1993). The psychological impact of infertility: a comparison with patients with other medical conditions. J. Psychosom. Obstet. Gynaecol. 14, 45–52. 8142988

[B8] Fard T. R. Kalantarkousheh M. Faramarzi M. (2018). Effect of mindfulness-based cognitive infertility stress therapy on psychological well-being of women with infertility. Middle East Fertil. Soc. J. 23, 476–481. 10.1016/j.mefs.2018.06.001

[B9] Gameiro S. Boivin J. Dancet E. De Klerk C. Emery M. Lewis-Jones C.. (2015). ESHRE guideline: routine psychosocial care in infertility and medically assisted reproduction - a guide for fertility staff. Hum. Reprod. 30, 2476–2485. 10.1093/humrep/dev177 26345684

[B10] Gameiro S. Boivin J. Peronace L. Verhaak C. M. (2012). Why do patients discontinue fertility treatment? A systematic review of reasons and predictors of discontinuation in fertility treatment. Hum. Reprod. Update 18, 652–669. 10.1093/humupd/dms031 22869759 PMC3461967

[B11] Gameiro S. Canavarro M. Boivin J. (2013). Patient centred care in infertility health care: direct and indirect associations with wellbeing during treatment. Patient Educ. Couns. 93, 646–654. 10.1016/j.pec.2013.08.015 24007764

[B12] Greiner A. L. Conklin J. (2015). Breaking bad news to a pregnant woman with a fetal abnormality on ultrasound. Obstet. Gynecol. Surv. 70, 39–44. 10.1097/OGX.0000000000000149 25616346

[B13] Groh C. J. Wagner C. (2005). The art of communicating ART results: an analysis of infertile couples' experience. J. Reprod. Infant Psychol. 23, 333–346. 10.1080/02646830500273533

[B14] Istituto Superiore di Sanità-ISS (2017). Available online at: https://www.iss.it/infertilità-e-pma (accessed October 03, 2020).

[B15] Jafarzadeh-Kenarsari F. Ghahiri A. Habibi M. Zargham-Boroujeni A. (2015). Exploration of infertile couples' support requirements: a qualitative study. Int. J. Fertil. Steril. 9, 81–92. 10.22074/ijfs.2015.4212 25918596 PMC4410041

[B16] JBI Critical Appraisal Checklist for Qualitative Research (2020). Available online at: https://joannabriggs.org/sites/default/files/2020-08/Checklist_for_Qualitative_Research.pdf (accessed December 01, 2020).

[B17] Klitzman R. (2018). Impediments to communication and relationships between infertility care providers and patients. BMC Womens Health. 18:84. 10.1186/s12905-018-0572-6 29871622 PMC5989459

[B18] Lalos A. (1999). Breaking bad news concerning fertility. Hum. Reprod. 14, 581–585. 10.1093/humrep/14.3.581 10221678

[B19] Leite R. C. Makuch M. Y. Petta C. A. Morais S. S. (2005). Women's satisfaction with physicians' communication skills during an infertility consultation. Patient Educ. Couns. 59, 38–45. 10.1016/j.pec.2004.09.006 16198217

[B20] Leone D. Menichetti J. Barusi L. Chelo E. Costa M. De Lauretis L.. (2017). Breaking bad news in assisted reproductive technology: a proposal for guidelines. Reprod. Health. 14:87. 10.1186/s12978-017-0350-1 28728610 PMC5520370

[B21] Liu W. (2015). Infertile patients' preference for receiving clinical information and participating in decision-making in China. Hum. Reprod. 30:347.

[B22] Mascarenhas M. N. Flaxman S. R. Boerma T. Vanderpoel S. Stevens G. A. (2012). National, regional, and global trends in infertility prevalence since 1990: a systematic analysis of 277 health surveys. PLoS Med. 9:e1001356. 10.1371/journal.pmed.1001356 23271957 PMC3525527

[B23] McBain T. D. Reeves P. (2019). Women's experience of infertility and disenfranchised grief. Fam. J. 27, 156–166 10.1177/1066480719833418

[B24] Moher D. Liberati A. Tetzlaff J. Altman D. G. Altman D. Antes G.. (2009). Preferred reporting items for systematic reviews and meta-analyses: The PRISMA statement. PLoS Med. 6:e1000097. 10.1371/journal.pmed.1000097 19621072 PMC2707599

[B25] Mosconi L. Ravaldi C. Vannacci A. (2019). A guideline to breaking bad news in prenatal ultrasound screening, in International Stillbirth Alliance, Annual Conference on Perinatal Mortality and Bereavement Care (Madrid). Available online at: https://www.isa2019madrid.com/breaking-bad-news-during-prenatal-ultrasound-screening/ (accessed December 1, 2020).

[B26] Palmer-Wackerly A. L. Voorhees H. L. D'Souza S. Weeks E. (2019). Infertility patient-provider communication and (dis)continuity of care: An exploration of illness identity transitions. Patient Educ. Couns. 102, 804–809. 10.1016/j.pec.2018.12.003 30527731

[B27] Peterson B. Boivin J. Norré J. Smith C. Thorn P. Wischmann T. (2012). An introduction to infertility counseling: A guide for mental health and medical professionals. J. Assist. Reprod. Genet. 29, 243–248. 10.1007/s10815-011-9701-y 22290604 PMC3288135

[B28] Quinn G. P. Bowman Curci M. Reich R. R. Gwede C. K. Meade C. D. Vadaparampil S. T. (2019). Impact of a web-based reproductive health training program: ENRICH (educating nurses about reproductive issues in cancer healthcare). Psychooncology 28, 1096–1101. 10.1002/pon.5063 30882960 PMC6597246

[B29] Stammer H. Wischmann T. Verres R. (2002). Counseling and couple therapy for infertile couples. Fam. Process. 41, 111–122. 10.1111/j.1545-5300.2002.40102000111.x 11924079

[B30] Streisfield A. Chowdhury N. Cherniak R. Shapiro H. (2015). Patient centered infertility care: The health care provider's perspective. Patient Exp. J. 2, 93–97 10.35680/2372-0247.1062

[B31] Taheri Z. Zeinalzadeh M. Ghanbarpour F. Taheri M. (2013). Effect of psychotherapy with acceptance and commitment therapy approach on reduction of infertility stress and increase of infertile couples' intimacy. Int. J. Fertil. Steril. 7(Suppl. 1), 157–158.

[B32] Ussher J. M. Parton C. Perz J. (2018). Need for information, honesty and respect: patient perspectives on health care professionals communication about cancer and fertility. Reprod. Health 15:2. 10.1186/s12978-017-0441-z 29304873 PMC5756327

[B33] Van den Broeck U. Emery M. Wischmann T. Thorn P. (2010). Counselling in infertility: Individual, couple and group interventions. Patient Educ. Couns. 81, 422–428. 10.1016/j.pec.2010.10.009 21075589

[B34] Van Empel I. W. H. Dancet E. A. F. Koolman X. H. E. Nelen W. L. D. M. Stolk E. A. Sermeus W.. (2011). Physicians underestimate the importance of patient-centredness to patients: A discrete choice experiment in fertility care. Hum. Reprod. 26, 584–593. 10.1093/humrep/deq389 21227936

