# Supplementary material for: Communication of Diagnosis of Infertility: A Systematic Review
Source: Front Psychol. 2021 Mar 18;12:615699. doi: 10.3389/fpsyg.2021.615699 (PMC8015870; doi:10.3389/fpsyg.2021.615699)
Supplement: Supplementary file 1 [file Table_1.DOCX]

***Supplementary Material***

1. **Quality of included studies according to the Checklist for Qualitative Research by Joanna Briggs Institute (JBI)**

**Author: Dancet et al.
Year: 2012
Record number: 10.1093/humrep/des061**

| **Item** | **Question** | **Yes** | **No** | **Unclear** | **NA** |
| --- | --- | --- | --- | --- | --- |
| 1 | Is there congruity between the stated philosophical perspective and the research methodology? | **X** |  |  |  |
| 2 | Is there congruity between the research methodology and the research question or objectives? | **X** |  |  |  |
| 3 | Is there congruity between the research methodology and the methods used to collect data? | **X** |  |  |  |
| 4 | Is there congruity between the research methodology and the representation and analysis of data? | **X** |  |  |  |
| 5 | Is there congruity between the research methodology and the interpretation of results? | **X** |  |  |  |
| 6 | Is there a statement locating the researcher culturally or theoretically? |  | **X** |  |  |
| 7 | Is the influence of the researcher on the research, and vice- versa, addressed? |  | **X** |  |  |
| 8 | Are participants, and their voices, adequately represented? | **X** |  |  |  |
| 9 | Is the research ethical according to current criteria or, for recent studies, and is there evidence of ethical approval by an appropriate body? | **X** |  |  |  |
| 10 | Do the conclusions draw in the research report flow from the analysis, or interpretation, of the data? | **X** |  |  |  |

**NA: not applicable**

**Author: Jafarzadeh-Kenarsari et al.
Year: 2015
Record number: 10.22074/ijfs.2015.4212**

| **Item** | **Question** | **Yes** | **No** | **Unclear** | **NA** |
| --- | --- | --- | --- | --- | --- |
| 1 | Is there congruity between the stated philosophical perspective and the research methodology? | **X** |  |  |  |
| 2 | Is there congruity between the research methodology and the research question or objectives? | **X** |  |  |  |
| 3 | Is there congruity between the research methodology and the methods used to collect data? | **X** |  |  |  |
| 4 | Is there congruity between the research methodology and the representation and analysis of data? | **X** |  |  |  |
| 5 | Is there congruity between the research methodology and the interpretation of results? | **X** |  |  |  |
| 6 | Is there a statement locating the researcher culturally or theoretically? |  |  | **X** |  |
| 7 | Is the influence of the researcher on the research, and vice- versa, addressed? |  |  |  | **X** |
| 8 | Are participants, and their voices, adequately represented? | **X** |  |  |  |
| 9 | Is the research ethical according to current criteria or, for recent studies, and is there evidence of ethical approval by an appropriate body? | **X** |  |  |  |
| 10 | Do the conclusions draw in the research report flow from the analysis, or interpretation, of the data? | **X** |  |  |  |

**NA: not applicable**

**Author: Liu et al.
Year: 2015
Record number**: Embase Identification Number L72005882

| **Item** | **Question** | **Yes** | **No** | **Unclear** | **NA** |
| --- | --- | --- | --- | --- | --- |
| 1 | Is there congruity between the stated philosophical perspective and the research methodology? | **x** |  |  |  |
| 2 | Is there congruity between the research methodology and the research question or objectives? | **x** |  |  |  |
| 3 | Is there congruity between the research methodology and the methods used to collect data? | **x** |  |  |  |
| 4 | Is there congruity between the research methodology and the representation and analysis of data? | **x** |  |  |  |
| 5 | Is there congruity between the research methodology and the interpretation of results? | **x** |  |  |  |
| 6 | Is there a statement locating the researcher culturally or theoretically? |  | **x** |  |  |
| 7 | Is the influence of the researcher on the research, and vice- versa, addressed? |  | **x** |  |  |
| 8 | Are participants, and their voices, adequately represented? | **x** |  |  |  |
| 9 | Is the research ethical according to current criteria or, for recent studies, and is there evidence of ethical approval by an appropriate body? | **x** |  |  |  |
| 10 | Do the conclusions draw in the research report flow from the analysis, or interpretation, of the data? |  |  | **X** |  |

**NA: not applicable**

1. **Quality of included studies according to the Checklist for the Quality Assessment of Guidelines (AGREE II)**

**Author: Gameiro et al.
Year: 2015
Record number: 10.1093/humrep/dev177**

| **tem** | **Score^*^** |
| --- | --- |
| **Domain 1: Scope and Purpose** |  |
| 1. The overall objective(s) of the guideline is (are) specifically described. | 7 |
| 2. The health question(s) covered by the guideline is (are) specifically described. | 7 |
| 3. The population (patients, public, etc.) to whom the guideline is meant to apply is specifically described | 7 |
| **Domain 2: Stakeholder Involvement** |  |
| 4. The guideline development group includes individuals from all relevant professional groups. | 7 |
| 5. The views and preferences of the target population (patients, public, etc.) have been sought. | 5 |
| 6. The target users of the guideline are clearly defined. | 7 |
| **Domain 3: Rigour of Development** |  |
| 7. Systematic methods were used to search for evidence. | 7 |
| 8. The criteria for selecting the evidence are clearly described. | 6 |
| 9. The strengths and limitations of the body of evidence are clearly described. | 6 |
| 10. The methods for formulating the recommendations are clearly described. | 6 |
| 11. The health benefits, side effects, and risks have been considered in formulating the recommendations. | 5 |
| 12. There is an explicit link between the recommendations and the supporting evidence. | 7 |
| 13. The guideline has been externally reviewed by experts prior to its publication. | Unclear |
| 14. A procedure for updating the guideline is provided. | 5 |
| **Domain 4: Clarity of Presentation** |  |
| 15. The recommendations are specific and unambiguous | 7 |
| 16. The different options for management of the condition or health issue are clearly presented. | 7 |
| 17. Key recommendations are easily identifiable. | 7 |
| **Domain 5: Applicability** |  |
| 18. The guideline describes facilitators and barriers to its application. | 3 |
| 19. The guideline provides advice and/or tools on the recommendations can be put into practice. | 7 |
| 20. The potential source implications of applying the recommendations have been considered. | 3 |
| 21. The guideline presents monitoring and/or auditing criteria. | 1 |
| **Domain 6: Editorial Independence** |  |
| 22. The views of the funding body have not influenced the content of the guideline. | NA |
| 23. Competing interests of guideline development group members have been recorded and addressed | NA |
| Rate the overall quality of this guideline | 6 |
| I would recommend this guideline for use (Yes; Yes, with modifications; No) | Yes |

***** Score of 1 for lowest possible quality to 7 for highest possible quality
