# Supplementary material for: Communication of Diagnosis of Infertility: A Systematic Review
Source: Front Psychol. 2021 Mar 18;12:615699. doi: 10.3389/fpsyg.2021.615699 (PMC8015870; doi:10.3389/fpsyg.2021.615699)
Supplement: Supplementary file 3 [file Table_3.DOCX]

***Supplementary Material***

1. **Search strategies for MEDLINE, Embase, PsycINFO, CINHAHL, and Psychology and Behavioral Sciences Collection (PBSC)**

***Healthcare professionals***

**MEDLINE**

1. ((infertil*[tiab] OR "Infertility"[Mesh] OR "Infertility, Male"[Mesh] OR "Infertility, Female"[Mesh] OR fertility[tiab] OR infertility[tiab] OR "reproductive health"[tiab]) AND (care[tiab] OR Patient-Centered Care[Mesh] OR counsel*[tiab] OR psychol*[tiab] OR psych*[tiab] OR communication[tiab] OR "Communication"[Mesh] OR "Communication Barriers"[Mesh] OR "Communication Methods, Total"[Mesh] OR "Nonverbal Communication"[Mesh] OR "Health Communication"[Mesh] OR "breaking bad news"[tiab] OR "bad news"[tiab] OR "communication diagnosis"[tiab] OR "Physician-Patient Relations"[Mesh]))
2. ("Nursing Staff, Hospital"[Mesh] OR "Nursing Staff"[Mesh] OR nurse*[tiab] OR "nursing staff"[tiab] OR "Medical Staff, Hospital"[Mesh] OR "Medical Staff"[Mesh] OR healthcare provider*[tiab] OR healthcare profession*[tiab] OR "Obstetric Nursing"[Mesh] OR obstetric*[tiab] OR "Midwifery"[Mesh] OR "Nurse Midwives"[Mesh] OR midwifery[tiab] OR midwives[tiab])
3. ("Adaptation, Psychological"[Mesh] OR "coping"[tiab] OR "coping strategies"[tiab] OR oncol*[tiab] OR cancer*[tiab] OR carcinom*[tiab] OR neoplas*[tiab] OR tumor[tiab] OR tumoral[tiab] OR tumorigen*[tiab] OR malignan*[tiab] OR oncogen*[tiab] OR mutagen*[tiab] OR oncolog*[tiab] OR "neoplasms"[Mesh] OR "Sexually Transmitted Diseases, Bacterial"[Mesh] OR "Sexual Behavior"[Mesh] OR "Adolescent"[Mesh] OR "Sex Education"[Mesh])
4. (case reports[ptyp] OR comment[sb] OR french[lang] OR spanish[lang] OR german[lang] OR chinese[lang] OR arabic[lang] OR italian[lang] OR turkish[lang] OR swedish[lang] OR danish[lang])
5. #1 AND #2
6. #5 NOT #3 NOT #4

**EMBASE**

1. (('infertility'/exp OR 'female infertility'/exp OR 'male infertility'/exp OR infertil*:ti,ab OR infertility:ti,ab OR 'reproductive health'/exp) AND (care:ti,ab OR 'care and caring'/exp OR 'gynecologic care'/exp OR 'patient care'/exp OR Patient-Centered Care:ti,ab OR counsel*:ti,ab OR psychol*:ti,ab OR psych*:ti,ab OR communication:ti,ab OR 'communication barrier'/exp OR 'total communication'/exp OR 'nonverbal communication'/exp OR 'medical information'/exp OR "breaking bad news":ti,ab OR "bad news":ti,ab OR "communication diagnosis":ti,ab OR 'doctor patient relationship'/exp))
2. 'nursing staff'/exp OR nurse*:ti,ab OR "nursing staff":ti,ab OR 'medical staff'/exp OR healthcare provider:ti,ab OR healthcare profession*:ti,ab OR 'obstetrical nursing'/exp OR obstetric*:ti,ab OR 'midwife'/exp OR 'nurse midwife'/exp OR midwifery:ti,ab OR midwives:ti,ab
3. 'coping behavior'/exp OR "coping":ti,ab OR "coping strategies":ti,ab OR cancer*:ti,ab OR carcinom*:ti,ab OR neoplas*:ti,ab OR tumor*:ti,ab OR tumoral*:ti,ab OR tumorigen*:ti,ab OR malignan*:ti,ab OR oncogen*:ti,ab OR mutagen*:ti,ab OR oncolog*:ti,ab OR 'neoplasm'/exp OR 'sexually transmitted disease'/exp OR 'sexual behavior'/exp OR 'adolescents'/exp OR 'sexual education'/exp
4. ([article]/lim OR [article in press]/lim OR [french]/lim OR [spanish]/lim OR [german]/lim OR [chinese]/lim OR [arabic]/lim OR [italian]/lim OR [turkish]/lim OR [swedish]/lim OR [danish]/lim)
5. #1 AND #2
6. #5 NOT #3 NOT #4

**PsycINFO, CINHAHL, and Psychology and Behavioral Sciences Collection (PBSC)**

1. (TI Infertility OR AB Infertility OR TI infertil* OR AB infertil* OR DE "Infertility" OR TI fertility OR AB fertility OR DE "Male Genital Disorders" OR DE "Reproductive Health" OR TI "female infertility" OR AB "female inefrtility") AND (TI care OR AB care OR TI "Patient-Centered Care" OR AB "Patient-Centered Care" OR TI counsel* OR AB counsel* OR TI psychol* OR AB psychol* OR TI psych* OR AB psych* OR TI communication OR AB communication OR DE "Communication" OR DE "Communication Barriers" OR TI "Communication Methods" OR AB "Communication Methods" OR DE "Interpersonal Communication" OR DE "Nonverbal Communication" OR TI "breaking bad news" OR AB "breaking bad news" OR TI "bad news" OR AB "bad news" OR DE "Medical Diagnosis" OR TI "diagnosis communication" OR AB "diagnosis communication" OR DE "Therapeutic Processes")
2. DE "Nursing" OR TI nurse* OR AB nurse* OR TI "nursing staff" OR AB "nursing staff" OR TI "Medical Staff" OR AB "Medical Staff " OR TI healthcare provider* OR AB healthcare provider* OR TI healthcare profession* OR AB healthcare profession* OR DE "Obstetricians" OR DE "Obstetrics" OR TI obstetric* OR AB obstetric* OR TI "Obstetric Nursing" OR AB "Obstetric Nursing" OR DE "Midwifery" OR TI midwifery OR AB midwifery OR TI midwives OR AB midwives OR AB "Nurse Midwives" OR TI "Nurse Midwives" OR AB "Nurse midwifery" OR TI "Nurse midwifery"
3. DE "Adaptation" OR DE "Adaptive Behavior" OR DE "Emotional Adjustment" OR DE "Coping Behavior" OR AB coping OR TI coping OR AB "coping strategies" OR TI "coping strategies" OR AB oncol* OR TI oncol* OR AB cancer* OR TI cancer* OR AB carcinom* OR TI carcinom* OR AB neoplas* OR TI neoplas* OR AB tumor* OR TI tumor* OR AB tumoral OR TI tumoral OR AB tumorigen* OR TI tumorigen* OR AB malignan* OR TI malignan* OR AB oncogen* OR TI oncogen* OR TI mutagen* OR AB mutagen* OR TI oncolog* OR AB oncolog* OR DE "Neoplasms" OR DE "Sexually Transmitted Diseases" OR DE "Sex Education" OR AB adolescent* OR TI adolescent* OR DE "Adolescent Attitudes" OR DE "Adolescent Behavior" OR DE "Psychosexual Behavior"
4. #1 AND #2
5. #4 NOT #3

***Patients***

**MEDLINE**

1. ((infertil*[tiab] OR "Infertility"[Mesh] OR "Infertility, Male"[Mesh] OR "Infertility, Female"[Mesh] OR fertility[tiab] OR infertility[tiab] OR "reproductive health"[tiab]) AND (care[tiab] OR Patient-Centered Care[Mesh] OR counsel*[tiab] OR psychol*[tiab] OR psych*[tiab] OR communication[tiab] OR "Communication"[Mesh] OR "Communication Barriers"[Mesh] OR "Communication Methods, Total"[Mesh] OR "Nonverbal Communication"[Mesh] OR "Health Communication"[Mesh] OR "breaking bad news"[tiab] OR "bad news"[tiab] OR "communication diagnosis"[tiab] OR "Physician-Patient Relations"[Mesh]))
2. (patient*[tiab] OR "Patients"[Mesh] OR "Women"[Mesh] OR "Pregnant Women"[Mesh] OR couple*[tiab])
3. ("Adaptation, Psychological"[Mesh] OR "coping"[tiab] OR "coping strategies"[tiab] OR oncol*[tiab] OR cancer*[tiab] OR carcinom*[tiab] OR neoplas*[tiab] OR tumor[tiab] OR tumoral[tiab] OR tumorigen*[tiab] OR malignan*[tiab] OR oncogen*[tiab] OR mutagen*[tiab] OR oncolog*[tiab] OR "neoplasms"[Mesh] OR "Sexually Transmitted Diseases, Bacterial"[Mesh] OR "Sexual Behavior"[Mesh] OR "Adolescent"[Mesh] OR "Sex Education"[Mesh])
4. (case reports[ptyp] OR comment[sb] OR french[lang] OR spanish[lang] OR german[lang] OR chinese[lang] OR hindi[lang] OR arabic[lang] OR italian[lang] OR turkish[lang] OR swedish[lang] OR danish[lang])
5. #1 AND #2
6. #5 NOT #3 NOT #4

**EMBASE**

1. (('infertility'/exp OR 'female infertility'/exp OR 'male infertility'/exp OR infertil*:ti,ab OR infertility:ti,ab OR 'reproductive health'/exp) AND (care:ti,ab OR 'care and caring'/exp OR 'gynecologic care'/exp OR 'patient care'/exp OR Patient-Centered Care:ti,ab OR counsel*:ti,ab OR psychol*:ti,ab OR psych*:ti,ab OR communication:ti,ab OR 'communication barrier'/exp OR 'total communication'/exp OR 'nonverbal communication'/exp OR 'medical information'/exp OR "breaking bad news":ti,ab OR "bad news":ti,ab OR "communication diagnosis":ti,ab OR 'doctor patient relationship'/exp))
2. 'patient'/exp OR 'obstetric patient'/exp OR patient*:ti,ab OR 'female'/exp OR 'pregnant woman'/exp OR 'couples'/exp OR 'couple'/exp OR couple*:ti,ab
3. 'coping behavior'/exp OR "coping":ti,ab OR "coping strategies":ti,ab OR cancer*:ti,ab OR carcinom*:ti,ab OR neoplas*:ti,ab OR tumor*:ti,ab OR tumoral*:ti,ab OR tumorigen*:ti,ab OR malignan*:ti,ab OR oncogen*:ti,ab OR mutagen*:ti,ab OR oncolog*:ti,ab OR 'neoplasm'/exp OR 'sexually transmitted disease'/exp OR 'sexual behavior'/exp OR 'adolescents'/exp OR 'sexual education'/exp
4. ([article]/lim OR [article in press]/lim OR [french]/lim OR [spanish]/lim OR [german]/lim OR [chinese]/lim OR [arabic]/lim OR [italian]/lim OR [turkish]/lim OR [swedish]/lim OR [danish]/lim)
5. #1 AND #2
6. #5 NOT #3 NOT #4

**PsycINFO, CINHAHL, and Psychology and Behavioral Sciences Collection (PBSC)**

1. (TI Infertility OR AB Infertility OR TI infertil* OR AB infertil* OR DE "Infertility" OR TI fertility OR AB fertility OR DE "Male Genital Disorders" OR DE "Reproductive Health" OR TI "female infertility" OR AB "female inefrtility") AND (TI care OR AB care OR TI "Patient-Centered Care" OR AB "Patient-Centered Care" OR TI counsel* OR AB counsel* OR TI psychol* OR AB psychol* OR TI psych* OR AB psych* OR TI communication OR AB communication OR DE "Communication" OR DE "Communication Barriers" OR TI "Communication Methods" OR AB "Communication Methods" OR DE "Interpersonal Communication" OR DE "Nonverbal Communication" OR TI "breaking bad news" OR AB "breaking bad news" OR TI "bad news" OR AB "bad news" OR DE "Medical Diagnosis" OR TI "diagnosis communication" OR AB "diagnosis communication" OR DE "Therapeutic Processes")
2. TI patient* OR AB patient* OR DE "Patient History" OR DE "Human Females" OR TI couple* OR AB couple* OR TI Pregnant Women OR AB Pregnant Women
3. DE "Adaptation" OR DE "Adaptive Behavior" OR DE "Emotional Adjustment" OR DE "Coping Behavior" OR AB coping OR TI coping OR AB "coping strategies" OR TI "coping strategies" OR AB oncol* OR TI oncol* OR AB cancer* OR TI cancer* OR AB carcinom* OR TI carcinom* OR AB neoplas* OR TI neoplas* OR AB tumor* OR TI tumor* OR AB tumoral OR TI tumoral OR AB tumorigen* OR TI tumorigen* OR AB malignan* OR TI malignan* OR AB oncogen* OR TI oncogen* OR TI mutagen* OR AB mutagen* OR TI oncolog* OR AB oncolog* OR DE "Neoplasms" OR DE "Sexually Transmitted Diseases" OR DE "Sex Education" OR AB adolescent* OR TI adolescent* OR DE "Adolescent Attitudes" OR DE "Adolescent Behavior" OR DE "Psychosexual Behavior"

1. #1 AND #2
2. #4 NOT #3
